# Optimal Extent of Prophylactic Irradiation of Paraaortic Lymph Nodes in Patients with Uterine Cervical Cancer

**DOI:** 10.1371/journal.pone.0145158

**Published:** 2015-12-14

**Authors:** Jinhyun Choi, Hong In Yoon, Jeongshim Lee, Ki Chang Keum, Gwi Eon Kim, Yong Bae Kim

**Affiliations:** 1 Department of Radiation Oncology, Yonsei Cancer Center, Yonsei University, College of Medicine, Seoul, Korea; 2 Department of Pharmacology, Brain Korea 21 plus Project for Medical Science, Yonsei University College of Medicine, Seoul, Korea; 3 Department of Radiation Oncology, Jeju National University School of Medicine, Jeju, Korea; 4 Yonsei Song-Dang Institute for Cancer Research, Yonsei University College of Medicine, Seoul, Korea; Rajiv Gandhi Centre for Biotechnology, INDIA

## Abstract

**Purpose:**

The purpose of this study was to determine optimal extent of prophylactic irradiation of paraaortic lymph nodes (PALN) in patients with uterine cervical cancer who had metastatic pelvic LNs.

**Methods and Materials:**

We retrospectively evaluated 103 patients with cervical cancer and pelvic lymph node metastasis who were treated with prophylactic semi-extended field radiotherapy (SEFRT) between 1990 and 2012. The semi-extended field included PALN below the second lumbar spine with prescribed doses of 45 to 50 Gy. Survival outcomes were calculated using the Kaplan-Meier method, and acute and late toxicities were scored using the Radiation Therapy Oncology Group and European Organisation for Research and Treatment of Cancer toxicity criteria.

**Results:**

The median follow-up after SEFRT was 61 (range 5–296) months. Overall, 28 patients (27.2%) experienced treatment failures, which were classified as local in 8 patients (7.8%), regional in 8 patients (7.8%), and distant in 13 patients (12.6%). Of the regional failures, only two involved PALN failure around the renal artery or the renal hilum area at the upper margin of the semi-extended field. At 5 years, the overall survival was 82%. Grade 3 or higher acute gastrointestinal and genitourinary toxicities occurred in one and two patients, respectively. As a late toxicity, one patient developed grade 3 small bowel obstruction.

**Conclusion:**

Prophylactic SEFRT provided favorable outcomes with little acute or late gastrointestinal toxicity. For prophylaxis of PALN recurrences, upper part of PALN might not need to be included in patients with uterine cervical cancer and metastatic pelvic LNs.

## Introduction

Whole-pelvic radiotherapy used definitively with or without chemotherapy is a mainstay of standard treatment and improves locoregional disease control and overall survival (OS) in patients with cervical cancer [1, 2]. Metastasis to the lymph nodes (LNs) is one of the most important prognostic factors in cervical cancer [3–5]. Uterine cervical cancer tends to be localized to the pelvis and to undergo an orderly lymphatic spread to the pelvic, para-aortic, and supraclavicular LNs [6, 7]. When the pelvic LN is involved, the incidence of common iliac and/or para-aortic LN involvement can reach 50% [8, 9]. Even if no lymphadenopathy is found in the para-aortic area, micrometastatic disease at the next echelon of nodes should be eradicated as part of the management of patients with cervical cancer with pelvic LN involvement.

Extended field radiotherapy (EFRT) has been used with curative intent, either prophylactically or therapeutically, according to the involvement of the para-aortic LN (PALN). Patients often suffer from serious toxicities, however, including gastrointestinal toxicities and especially duodenal injury. To minimize those kinds of complications, our institutional policy for the last two decades has been to use semi-extended field radiotherapy (SEFRT) that excludes the upper one third of the PALN chain. The purpose of this study is to assess the clinical outcomes in patients treated with SEFRT and determine optimal extent of prophylactic irradiation of PALN in patients with uterine cervical cancer who had metastatic pelvic LNs.

## Materials and Methods

### Patient characteristics and treatment profiles

This retrospective study was approved by the Institutional Review Board of the Severance hospital (IRB No. 4-2015-0059). The consent was not necessary, because patient records and information were anonymized and de-identified prior to analysis. In this study, a total of 103 patients with International Federation of Gynecology and Obstetrics (FIGO) stage IB to stage IVA cervical cancer who were treated with SEFRT at Yonsei Cancer Center from 1990 to 2012 were retrospectively analyzed. All of the patients underwent a physical examination, pelvic examination, complete blood cell counts, and chemistry profiles including liver and renal function tests as a baseline study. LN metastases were evaluated by computed tomography [10], magnetic resonance imaging (MRI), positron emission tomography (PET), or PET-CT. LNs larger than 1 cm in the short-axis dimension were considered to have metastatic involvement. Additionally, we regarded central necrosis as a significant criterion for metastatic disease within the LN [11]. For the PET or PET-CT image interpretation, a malignant lymphadenopathy was defined as follows: 1) fluorodeoxyglucose accumulation in the LN greater than that in the liver or similar to that in the brain cortex or 2) a standardized uptake value of a lesion, which corresponded to the CT, that did not decrease on the delayed PET image compared with that on the initial PET image [12]. None of the patients had the PALN assessed surgically.

**Fig 1:**
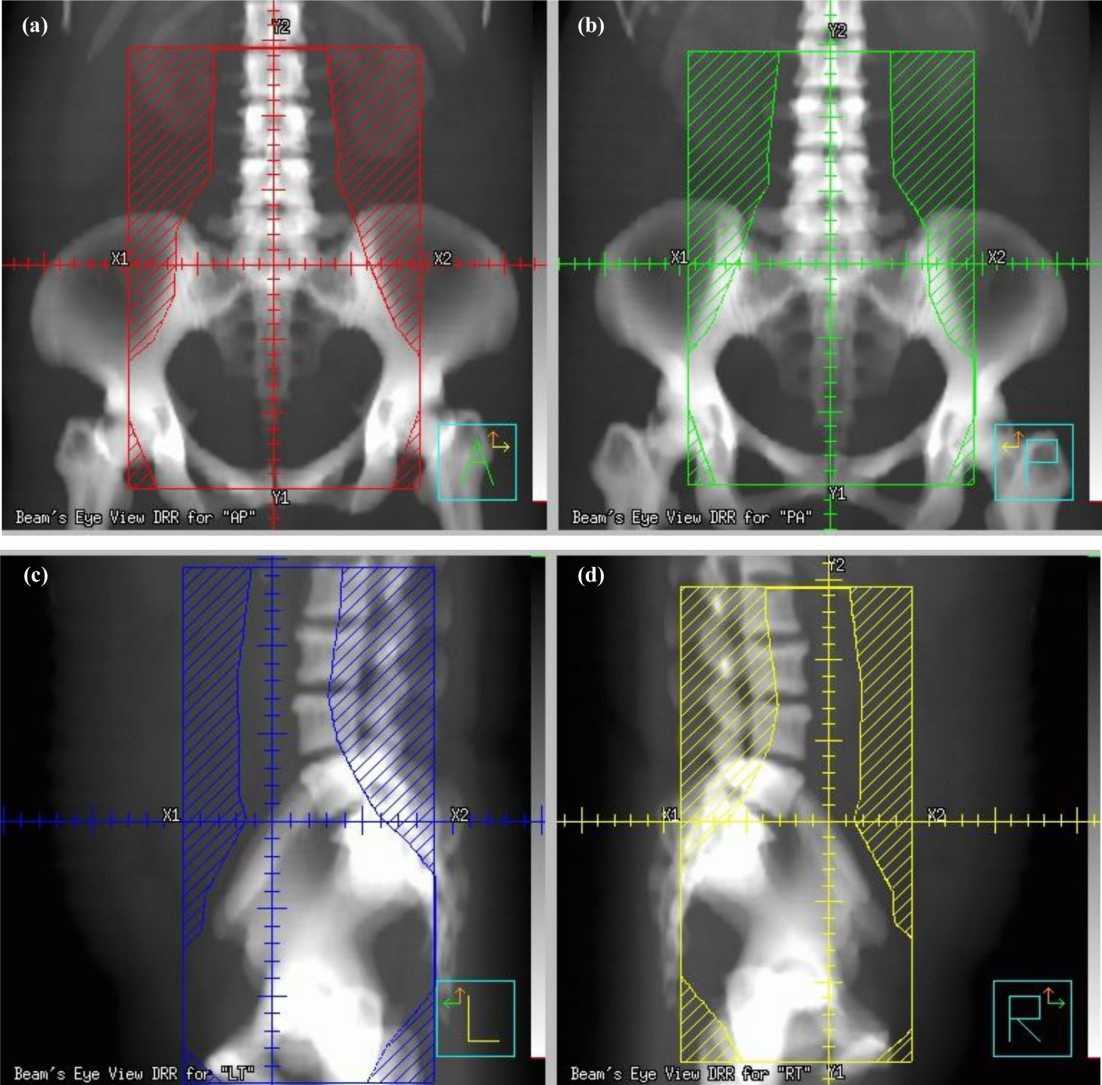
The beam’s eye view of the semi-extended radiation field using a four-field technique. (a) Anterior-Posterior, (b) Posterior-Anterior, (c) Left lateral, and (d) Right lateral.

In our institution, patients with cervical cancer received individualized RT according to pelvic and para-aortic nodal status as follows: whole-pelvic RT for negative LN, SEFRT to exclude upper PALN for positive pelvic LN only, and EFRT for PALN metastasis. SEFRT was delivered using a four-field (anterior-posterior/posterior-anterior and two lateral fields) box technique. The superior border was the second lumbar spine (L2), and the inferior border was the obturator foramen or at least 2 cm beyond the lower extent of the disease. The lateral border of the pelvis and the semi-extended field (SEF) encompassed areas 1.5 cm beyond the bony pelvic rim and 1 cm lateral to the aorta or tips of transverse processes, respectively (Fig 1). In the lateral view, the superior and inferior borders were identical to those of the anterior-posterior/posterior-anterior fields. The anterior border covered the symphysis pubis and intersected the posterior border at the S2-S3 space. The anterior border of the SEF was 2 cm anterior to the vertebral body surface.

We prescribed a radiation dose of 45 Gy in 25 fractions to the SEF, which is the same dose used in EFRT. Intracavitary high-dose rate brachytherapy consisted of 30–39 Gy in 6–13 fractions given two or three times per week. A midline block was added after 30.6–45 Gy during the insertions of intracavitary brachytherapy. Sites of pelvic LN metastases received an additional external boost to a median dose of 5.4 Gy (range: 5.4–18 Gy) at the discretion of the radiation oncologists.

### Assessment

Acute toxicities were evaluated at least once per week during treatment. After the completion of treatment, all patients were evaluated every three months for the first two years and every six months thereafter. Follow-up evaluations included a pelvic examination, cervical cytology, and CT or MRI. Late toxicities were evaluated from six months onward after treatment, and the development of gastrointestinal (rectum, small bowel) and urogenital symptoms was noted. Acute and late toxicities were scored using the Radiation Therapy Oncology Group (RTOG) and European Organisation for Research and Treatment of Cancer toxicity criteria [13].

### Statistical analysis

Patterns of failure, OS, and recurrence-free survival (RFS) were evaluated. The sites of failure were recorded as local, regional, or distant. In terms of the RT field, in-field failure was defined as disease in the pelvic area and the PALN within the SEF, and out-field failure was defined as disease outside the treatment field, especially in the PALN located at the superior border of L2 or higher. Survival was defined from the date of the completion of RT to the date of the last follow-up or death. Time to recurrence was measured from the date of completion of RT to the date of first failure. Survival data were analyzed using the Kaplan-Meier method. A univariate analysis was performed using the log-rank test to identify parameters associated with the treatment outcome, and multivariate analyses were performed using a Cox regression model. A p-value ≤ 0.05 was considered statistically significant.

## Results

### Patient characteristics

The median patient age was 52 years (range: 28–81 years). The characteristics of the patients are listed in Table 1. Most patients had FIGO stage IB to IIIB disease. The tumor histology was defined as squamous cell carcinoma in 98 patients and adenocarcinoma in four patients. Seventy-six patients (73.8%) received concurrent chemoradiation therapy, and 26 patients (25.2%) were treated with radiotherapy alone.

**Table 1:** Patient and tumor characteristics.

Variable		Level	Number of patients (%)
**All patients**			103 (100)
**Age, yr**		**Median (range)**	52 (28–81)
**FIGO stage**		**I**	11 (10.7)
		**II**	65 (63.1)
		**III**	26 (25.2)
		**IV**	1 (1.0)
**Histology**		**Squamous**	98 (95.1)
		**Adenocarcinoma**	4 (3.9)
		**Other**	1 (1.0)
**Tumor size, cm**		**Median(range)**	5 (1–9.7)
**Highest nodal involvement**		**Pelvic cavity**	24 (23.3)
		**Iliac chain**	79 (76.7)
**EBRT dose (cGy)**		**4500**	100 (97.1)
		**5040**	3 (2.9)
**LN boost (cGy)**		**540**	46 (44.7)
		**900**	34 (33.0)
		**>900**	5 (4.9)
		**None**	18 (17.5)
**ICR courses, fx**		**Median (range)**	6 (6–13)
**Point A total dose, cGy**		**Median (range)**	6600 (5340–10260)
**Chemotherapy**		**Concurrent**	76 (73.8)
		**Induction**	1 (1.0)
		**None**	26 (25.2)
**Overall treatment duration, wk**		**Median(range)**	9.6 (6.4–17.7)
**Follow-up, mo**		**Median(range)**	61 (5–296)

Abbreviations: FIGO = The International Federation of Gynecology and Obstetrics; EBRT = External Beam Radiotherapy; LN = lymph node; ICR = Intracavitary radiotherapy.

The treatment compliance was good, and all patients completed the scheduled RT, although there were delays of RT because of acute toxicity, mainly manifesting as hematologic problems, in 11 patients. The median duration of overall treatment was 9.6 weeks (range: 6.4–17.7 weeks).

### Treatment outcomes

**Fig 2:**
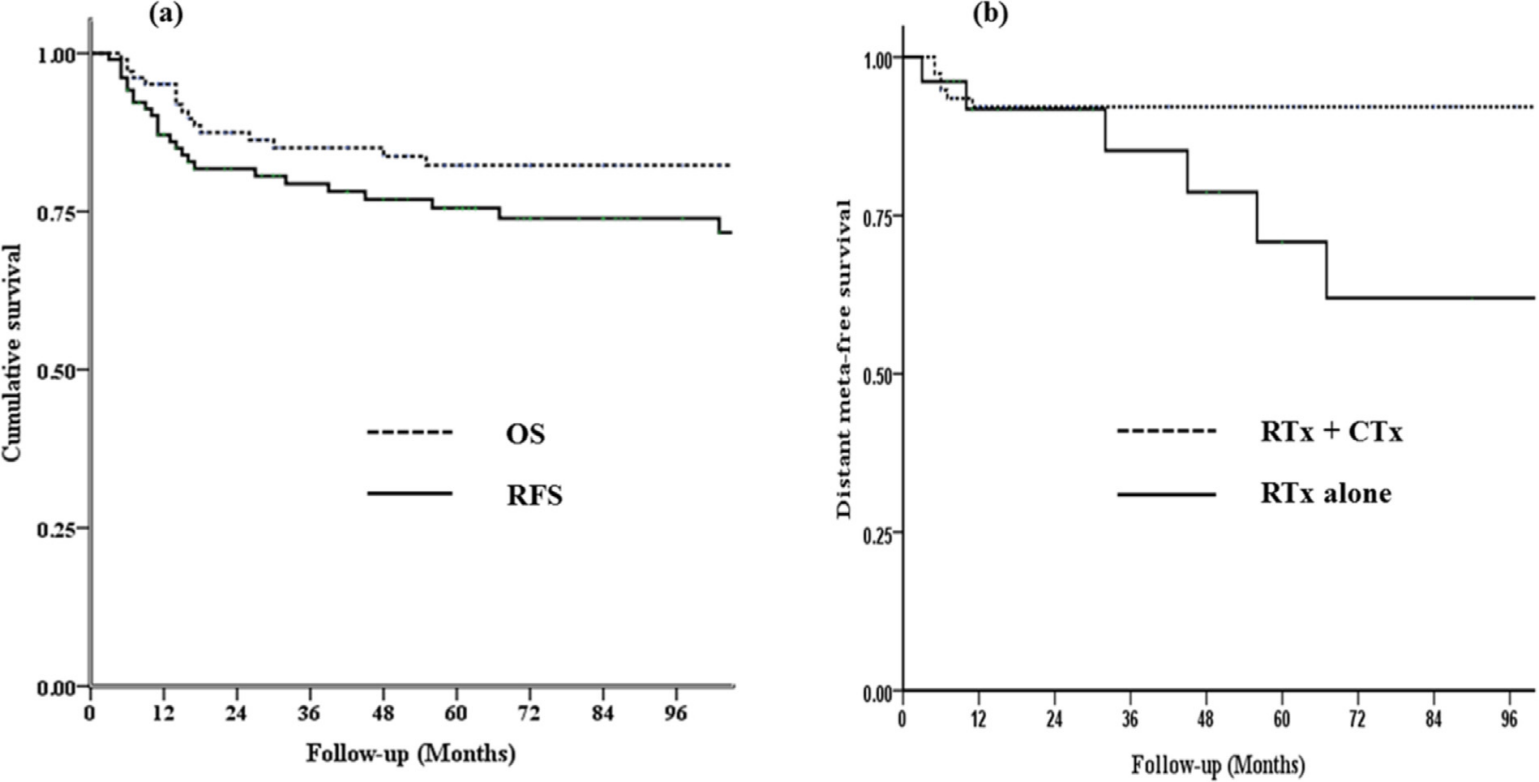
Kaplan-Meier survival curves for the 103 patients analyzed in this study. (a) Overall Survival (OS) and Recurrence Free Survival (RFS) rates, (b) Distant Metastasis-Free Survival (DMFS) rates for the treatment with radiotherapy (RTx) and chemotherapy (CTx) versus treatment with RT alone.

The median follow-up period for surviving patients was 61 months (range: 5–296 months). The five-year actuarial OS, RFS, local failure-free survival (LFFS), regional failure-free survival (RFFS) and distant metastasis-free survival (DMFS) rates were 82%, 76%, 94%, 92%, and 88%, respectively. The curves of the 5-year OS and RFS rates are shown in Fig 2. The results of the analyses of prognostic factors are presented in Table 2. The univariate analysis showed that the 5 year RFFS was significantly different between patients with stage I–II and stage III–IV disease, respectively (97% vs. 76%, p = 0.008). The use of chemotherapy was associated with improved 5-year DMFS (92% vs. 71%, p = 0.039; Fig 2). In case of iliac chain involvement, 5 year LFFS was higher than without it, for example, obturator nodes deep in the pelvis (96.9% vs. 85.9%, p = 0.02).

**Table 2:** Effect of prognostic factors on treatment outcomes in univariate analyses.

Prognostic factor	5-yr OS		5-yr RFS		5-yr LFFS		5-yr RFFS		5-yr DMFS	
	%	p	%	p	%	p	%	p	%	p
**Age**		0.14		0.446		0.217		0.687		0.856
**≤50yr**	77.5		76.4		92.4		92		90.9	
**>50yr**	85.9		74.5		96		91.7		85.3	
**Tumor size**		0.92		0.561		0.944		0.568		0.984
**≤4cm**	81.1		78.9		95.1		94.8		87.7	
**>4cm**	83.4		72.9		94		89.3		88	
**Stage**		0.415		0.118		0.342		0.008		0.441
**FIGO I-II**	85.3		81		96.8		96.9		86.5	
**FIGO III-IV**	73.3		59.7		87.6		76		92.6	
**Chemotherapy**		0.892		0.196		0.898		0.864		0.039
**Yes**	82.8		78.2		94		90.8		92.2	
**No**	80.8		64.8		96		96.2		70.8	
**Nodal involvement**		0.982		0.126		0.02		0.819		0.371
**Iliac chain(+)**	83		77.6		96.9		91.4		88.1	
**Iliac chain(-)**	78.7		69.2		85.9		92.9		87.5	
**RT duration**		0.708		0.88		0.659		0.816		0.836
**≤9wks**	86.4		76.2		97.1		92		85.6	
**>9wks**	79.2		75.3		92.6		91.7		89.5	

Abbreviations: OS = overall survival; RFS = relapse free survival; LFFS = local failure free survival; RFFS = regional failure free survival; DMFS = distant metastasis free survival; FIGO = The International Federation of Gynecology and Obstetrics

Ninety-five patients (92.2%) had complete remission, and eight patients (7.8%) had partial remission after the completion of RT. No stable or persistent disease was observed in any of the patients. During the follow-up period, 75 patients (72.8%) had no recurrence, and 28 patients (27.2%) experienced treatment failure as follows: eight patients (7.8%) had local recurrence, eight patients (7.8%) had regional recurrence, and 13 patients (12.6%) had distant metastasis at the time of analysis. One patient had simultaneous regional and distant recurrence.

**Fig 3:**
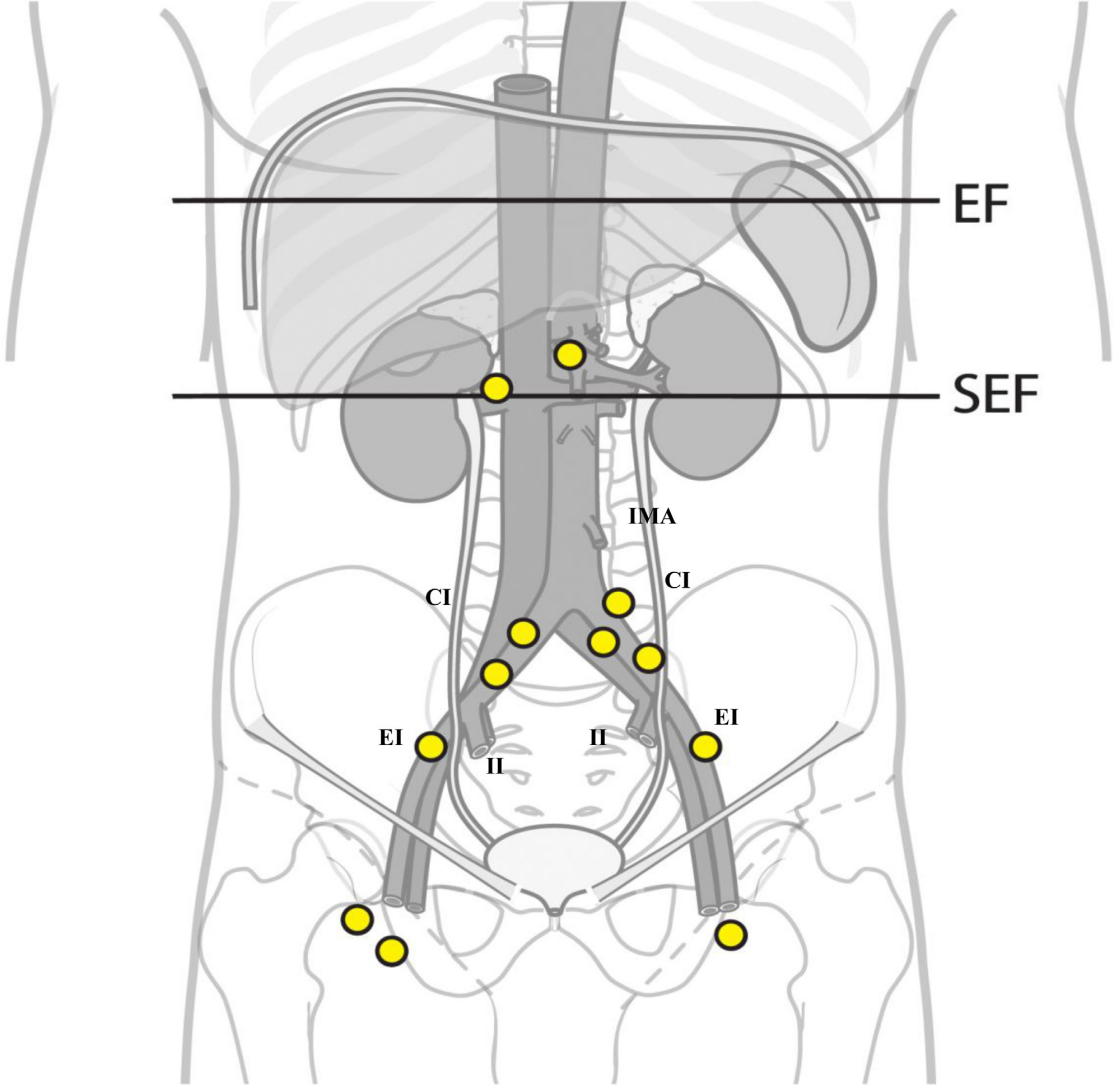
The patterns of nodal recurrence for seven patients. A total 10 recurrent sites are indicated along the inferior mesenteric artery (IMA), common iliac (CI), external iliac (EI), internal iliac (II) chain, and inguinal iliac chain. Two para-aortic LN failures lie between the upper borders of the semi-extended field (SEF) and the extended field (EF), respectively.

Of the regional failures, a total of four recurrences occurred exclusively within the SEF. Another four recurrences were found outside of the SEF. Fig 3 shows the patterns of nodal recurrences. PALN failures occurred in two patients (1.9%). One patient treated with SEFRT with a superior border at the L3 level had failure in both the PALN at the renal hilum level and the supraclavicular LN. Another patient had an isolated PALN failure at the renal artery level after concurrent chemoradiation therapy. The characteristics of the patients who had nodal failures are summarized in Table 3.

**Table 3:** Characteristics of patients with nodal failure.

No	Age	Stage	RTx (EBRT+ LN boost)	CTx	Initial LN involved	Nodal failure level	Interval (m)	Superior border of SEF
**1**	79	IIIA	45Gy+5.4Gy	None	Rt.external iliac	Both external iliac, inguinal	5	L2
**2**	47	IIIB	45Gy+5.4Gy	Concurrent	Rt.external iliac	Lt. common iliac	11	L2
**3**	50	IIB	45Gy+9Gy	Concurrent	Rt.external iliac	Both common iliac	14	L2
**4**	41	IIB	45Gy+5.4Gy	Concurrent	Both external iliac and internal iliac	Both common iliac	16	L3
**5**	62	IIIB	45Gy+5.4Gy	Concurrent	Lt. external iliac	Rt.inguinal	9	L2
**6**	43	IIB	45Gy+none	None	Lt. obturator	Paraaortic (Renal hilum)	67	L3
**7**	63	IVA	45Gy+9Gy	Concurrent	Both hypogastric area	Paraaortic (Renal artery)	39	L2

Abbreviations: RTx = radiotherapy; EBRT = external beam radiotherapy; CTx = chemotherapy; LN = lymph node; SEF = semi-extended field

### Treatment-related toxicity

Most of the acute grade 3–4 toxicities were hematologic problems including anemia and neutropenia. Although RT for six patients (5.8%) was delayed because of low neutrophil counts, all of the patients were properly managed and recovered sufficiently to continue the scheduled treatment. Acute gastrointestinal toxicity was observed in 21 patients (20.4%), of which mild and moderate diarrhea and abdominal pain were the most common toxicities (grade 1 or 2). One patient experienced acute grade 3 diarrhea and received treatment without RT delay. No serious acute genitourinary toxicities were noted.

Fourteen patients (13.6%) suffered late gastrointestinal toxicities. Most of those were lower gastrointestinal problems located entirely in the pelvic field. One patient had a grade 2 partial small bowel obstruction, which was not surgically managed but required supportive care such as L-tube insertion. Three patients (2.9%) experienced late grade 3 toxicities. Among those, one patient had a small bowel obstruction that required surgical intervention at 158 months. The patient underwent an ileostomy and segmental resection of the small intestine. Other patients had radiation proctitis. Another patient was diagnosed with radiation proctitis but refused any evaluations and treatments except for transfusion to treat the severe anemia caused by 7 months of rectal bleeding. That patient expired 14 months after RT due to progressive rectal bleeding. As a genitourinary toxicity, two patients experienced vesicovaginal/recto-vesicovaginal fistulas that required surgical management at 11 months and 12 months, respectively. No patients experienced grade 4 or higher genitourinary toxicity.

## Discussion

Cervical cancer metastasizes to the regional LNs, spreading in a contiguous manner, initially involving the lower pelvis and then progressing to the high pelvic LNs, including the common iliac nodes followed by the para-aortic nodes [14]. Consequently, occult metastasis or micrometastasis to the common iliac and/or para-aortic LNs can occur in patients with pelvic LN involvement, and failures occur just outside the standard pelvic radiation field [15]. Those patients may benefit from EFRT to prevent recurrence at PALN chain.

A profound understanding the flow of the PALN is crucial for determining the extent of RT and improving patient survival, although the question of the extent of the optimal superior border of pelvic RT remains unresolved. Given the anatomy of lymphatic drainage, the right and left lumbar trunks formed by the union of the efferent vessels from the lateral aortic lymph glands receive the lymph from the common iliac chains, ovaries, uterine tubes, and body of the uterus. Ultimately, the lumbar trunks empty into the cisterna chyli, a dilated sac at the beginning of the thoracic duct that drains into the left subclavian vein [16, 17]. Therefore, the cisterna chyli located at the level of L2 seems to play an important role as a sort of station for systemic metastasis through the PALNs. Clinicopathological studies also provide a rationale for setting the superior border of the SEF at the L2 level [18]. On determining the surgical staging extent of the PALNs, some gynecological oncologists consider the origin of the inferior mesenteric artery (IMA) as the upper dissection margin based on the finding that PALN metastases above the IMA hardly occur in the absence of node metastasis below the IMA. According to a review of 733 complete infrarenal lymphadenectomies, 54 patients (54/207, 26.08%) had PALN metastasis with pelvic node metastasis. Of those with PALN metastasis, only 10 patients (1.36%) were found to have LN metastasis above the level of the IMA without node metastasis in the lower part of the para-aortic area [19]. Therefore, for patients who are positive only in the pelvic node, prophylactic irradiation to prevent PALN recurrence might be enough to cover the cisterna chyli and the level of the IMA up to L2.

Detailed patterns of regional failure can help determine extent of the radiation field. Researchers at the M.D. Anderson Cancer Center analyzed the relationship between recurrence and the radiation field and showed that the most common site of regional recurrence was marginal, usually just above the superior boundary of the RT field [20]. Of the 198 patients who had a regional recurrence after definitive RT for cervical cancer, 103 (52%) were treated with pelvic RT with a superior field border at L4/5, while 7 (3.5%) were treated with pelvic RT with a superior field border at L2/3. Of the 180 patients who had an evaluable regional recurrence, 119 (66%) had a component of marginal failure, indicating that the proportion of nodal recurrence may be higher with a smaller field. Thus, the study at M.D. Anderson provided strong support for our institutional practice of setting the upper border of the EF at the L2 spine level if bowel sparing could be established.

Several previous studies showed that patients with pelvic LN involvement were effectively treated with EFRT but that treatment-related toxicity was relatively severe. A prospective randomized study demonstrated that prophylactic EFRT with elective para-aortic irradiation improved survival and reduced distant metastases compared with pelvic-only irradiation [21]. The cumulative incidence of grade 4–5 toxicities after 10 years in the EFRT arm was 8% compared with 4% in the pelvic-only arm. Extending the radiation field to the para-aortic region increased the irradiated dose to the small bowel, especially the duodenum. In addition, a previous analysis showed EFRT to be a significant predictor of severity and chronicity of ongoing disease in patients who live with radiation-induced bowel injury after treatment for cervical cancer [22]. In this study, we hypothesized that using SEFRT to exclude the upper PALNs could reduce toxicities, especially upper gastrointestinal toxicities, without compromising treatment outcomes. Among the four patients who experienced grade 3 or higher gastrointestinal toxicity, only one experienced small bowel obstruction requiring surgical intervention. That encouraging finding supports the hypothesis that SEFRT might be considered for reducing upper gastrointestinal toxicities, although there has been no prospective clinical trial to evaluate the efficacy of SEFRT compared with that of EFRT until now.

SEFRT also showed favorable PALN control and survival outcomes. When the profiles of nodal failures were analyzed according to the radiation field, only two patients (1.9%) experienced out-field PALN failures between the respective upper borders of SEFRT and EFRT. Other failures were classified as in-field failures or distant nodal failures. The five-year OS rate in the EFRT arms was 67% and 52% in RTOG trial 79–20 and RTOG trial 90–01, respectively [21, 23]. Recent institutional series showed 5 year OS rates of 73.5% and 72.4%, respectively [24, 25]. The 5 year survival rate in our study was superior to those in the previous studies, suggesting that SEFRT excluding the upper PALN does not significantly compromise PALN control, survival outcomes, and toxicities compared with EFRT.

Intensity-modulated radiotherapy (IMRT) has been considered a promising modality to reduce toxicity and improve effective nodal control and treatment outcomes. A recent study reported that IMRT may allow sufficient dose sparing of the small bowel and was associated with no duodenal-specific toxicity for the treatment of the para-aortic nodes. The study reported on the clinical outcomes for 32 patients treated with prophylactic EF-IMRT [26]. The 3 year actuarial OS was 87% and only one patient experienced late grade 3 gastrointestinal complication. Because EF-IMRT was shown to be a safe and effective modality, SEF-IMRT is expected to have less toxicity with comparable treatment outcomes.

Our findings must be interpreted with caution because of several drawbacks. Because this study was a retrospective study covering over 20 years, heterogeneity among the patient characteristics might have confounded the results. The most important issue is how to evaluate metastasis of the pelvic and para-aortic LNs, which were determined not by laparotomy but by various imaging modalities including CT, MRI, PET, and PET-CT over a long period.

In conclusion, our results suggest that prophylactic SEFRT in patients with uterine cervical cancer and metastasis in the pelvic LNs without PALN involvement provided favorable treatment outcomes with acceptable acute and late gastrointestinal toxicities comparable to those of EFRT. Therefore upper part of PALN might not need to be included in patients with uterine cervical cancer and metastatic pelvic LNs for prophylaxis of PALN recurrences.
